# Metabolic Engineering of Model Microorganisms for the Production of Xanthophyll

**DOI:** 10.3390/microorganisms11051252

**Published:** 2023-05-09

**Authors:** Nan Wang, Huakang Peng, Caifeng Yang, Wenfang Guo, Mengqi Wang, Gangqiang Li, Dehu Liu

**Affiliations:** Biotechnology Research Institute, Chinese Academy of Agricultural Sciences, Beijing 100081, China

**Keywords:** xanthophyll, metabolic engineering, biosynthetic pathway, model microorganisms

## Abstract

Xanthophyll is an oxidated version of carotenoid. It presents significant value to the pharmaceutical, food, and cosmetic industries due to its specific antioxidant activity and variety of colors. Chemical processing and conventional extraction from natural organisms are still the main sources of xanthophyll. However, the current industrial production model can no longer meet the demand for human health care, reducing petrochemical energy consumption and green sustainable development. With the swift development of genetic metabolic engineering, xanthophyll synthesis by the metabolic engineering of model microorganisms shows great application potential. At present, compared to carotenes such as lycopene and β-carotene, xanthophyll has a relatively low production in engineering microorganisms due to its stronger inherent antioxidation, relatively high polarity, and longer metabolic pathway. This review comprehensively summarized the progress in xanthophyll synthesis by the metabolic engineering of model microorganisms, described strategies to improve xanthophyll production in detail, and proposed the current challenges and future efforts needed to build commercialized xanthophyll-producing microorganisms.

## 1. Introduction

**Figure 1:**
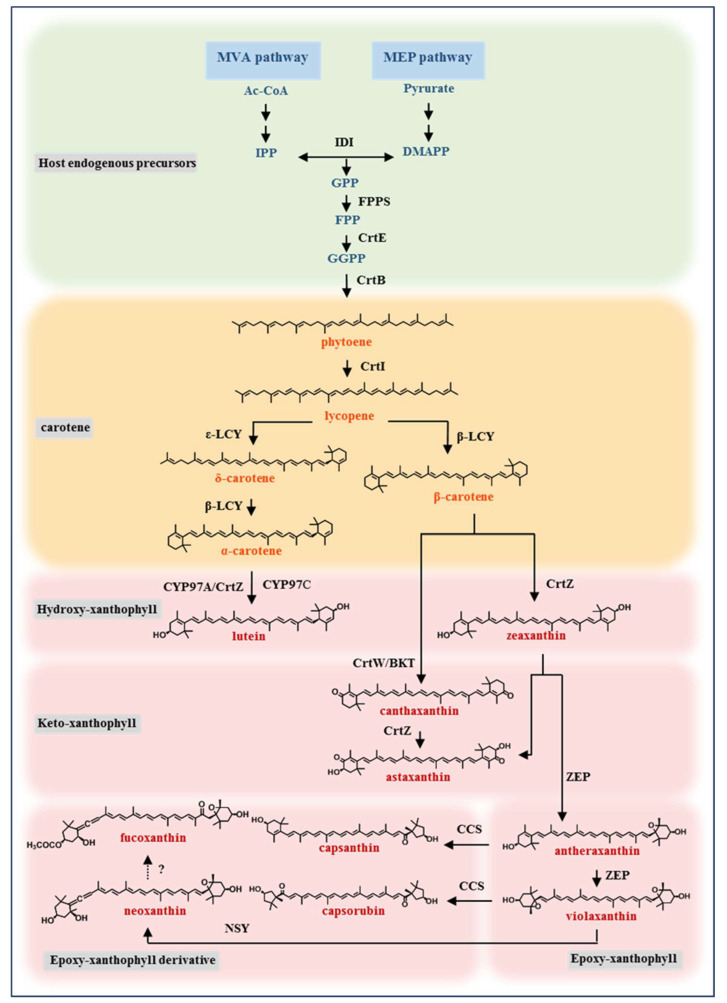
Xanthophyll biosynthesis pathway. Abbreviations: MVA, mevalonate; MEP, 2-C-methyl-D -erythritol-4-phosphate; IPP, isoprene diphosphate; DMAPP, dimethylallyl diphosphate; GPP, geranyl diphosphate; FPP, farnesyl diphosphate; GGPP, geranylgeranyl diphosphate; IDI, IPP isomerase; FPPS, farnesyl diphosphate synthase; CrtE, GGPP synthase; CrtB, phytoene synthase; CrtI, phytoene desaturase; ε-LCY, lycopene ε-cyclase; β-LCY, lycopene β-cyclase; CYP97A, α-carotene β-ring hydroxylase; CYP97C, α-carotene ε-ring hydroxylase; CrtZ, β-carotene hydrolase; CrtW, β-carotene ketolase (from bacteria); BKT, β-carotene ketolase (from algae); ZEP, zeaxanthin epoxidase; CCS, capsanthin/capsorubin synthase; NSY, neoxanthin synthase. The dotted line indicates that the enzyme catalyzing the reaction has not been identified.

Carotenoids are a class of secondary metabolites with a tetraterpene structure that are widely found in plants, algae, yeast, archaea, and some bacteria species [1,2,3,4,5]. Carotenoids are classified into two categories depending on whether they contain oxygen (Figure 1): carotene and xanthophyll [6,7]. The structure and function of xanthophyll present abundant diversity attributed to the oxygen group, which exists in various forms, such as hydroxyl-, keto-, expo-xanthophyll, and expo-xanthophyll derivatives [8]. So far, more than 600 xanthophylls (including isomers) have been found or identified among approximately 1000 carotenoids (https://coconut.naturalproducts.net/ (accessed on 3 March 2023).

Xanthophyll has been used commercially in a wide variety of industries, such as food, feed, pharmaceuticals, and cosmetics [8,9,10]. Currently, chemical processing and conventional extraction from natural organisms are the mainly source of xanthophyll. Despite the low cost and high yield of chemical synthesis methods, the formation of unnatural chiral isomer reduces the bioavailability of the product. In addition, petrochemical-refined xanthophyll is not allowed to be used in food and pharmaceutical products. The yield of xanthophyll extracted from plants or algae is too low to meet industrial needs [11,12]. In recent decades, metabolic engineering based on model microbes for the production of nature bioactivity compounds has experienced extensive development [13,14,15], which brings a promising potential for the industrial synthesis of natural xanthophyll.

The elucidation of the natural synthetic pathways and key enzymes of several important xanthophylls has laid the foundation for xanthophyll synthesis by the metabolic engineering of model microorganisms [16,17,18,19,20]. The natural synthetic pathway of xanthophyll is a cascade reaction that begins with the oxidative modification of the α- or β-carotenoid terminal group (Figure 1). The hydroxylation of α- and β-carotene terminal groups enables the production of lutein and zeaxanthin, respectively [9,21,22,23]. Lutein and zeaxanthin, two yellow hydroxyl-xanthophylls, are used to treat aging-related macular degeneration because they are the only and necessary carotenoids for the macular function of the human eye [2,21]. The β-carotene and zeaxanthin can be ketonylated to produce canthaxanthin and astaxanthin, two orange or red keto-xanthophylls [24,25]. They are used as feed additives in aquaculture and poultry farming to give fish or egg yolks a red color [1,11,26]. Astaxanthin is also widely used in nutraceuticals, pharmaceuticals, and cosmetics due to its excellent antioxidant activity [11,27]. Epoxy-xanthophyll, antheraxanthin, and violaxanthin are derived from another branch of the metabolic pathway of zeaxanthin [28]. The terminal epoxide groups of antheraxanthin and violaxanthin can be further modified to produce capsanthin, capsorubin, neoxanthin, etc. [29,30,31,32,33,34,35,36]. These different modifications allow xanthophyll to show a broader color variation and stronger antioxidant activity compared to carotene [24,37,38,39,40].

**Figure 2:**
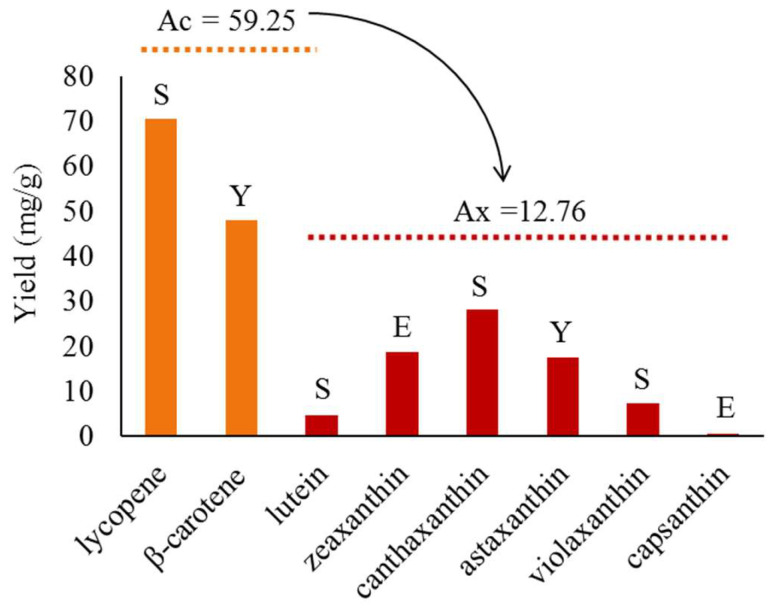
The average value of the highest yield of carotene (lycopene and β-carotene) and xanthophyll (lutein, zeaxanthin, canthaxanthin, astaxanthin, violaxanthin, and capsanthin) in engineering model microorganisms. “S” refers to *Saccharomyces cerevisiae*, “Y” refers to *Yarrowia lipolytica*, “E” refers to *Escherichia coli*.

The number of articles on xanthophyll metabolic engineering in non-natural microbial producers has increased rapidly in the last decade, and the highest yield of various xanthophyll has also continuously increased over time. However, in engineered microorganisms, the yield of xanthophyll was significantly lower than that of their precursor, such as lycopene and β-carotene [41,42]. Under shake flask culture conditions, the average value of the highest yield of lycopene and β-carotene reached 59.25 mg/g, whereas that of xanthophyll was only 12.76 mg/g according to the articles published so far (Table 1 and Figure 2).

Compared to carotene, xanthophyll has a longer synthetic pathway, resulting in a more complex microbial metabolic engineering; in addition, xanthophyll is more polarized, resulting in a more harmful effect on host cells [43,44]. These innate characteristics make xanthophyll production in an engineering host more difficult to improve. The lower production is the main bottleneck for the commercialization of xanthophyll synthesized by microbial metabolic engineering.

In this review, we focused on the research progress and strategies of the metabolic engineering of xanthophyll synthesis in model microorganisms, including *Escherichia coli*, *Saccharomyces cerevisiae*, *Yarrowia lipolytica*, *Pichia pastoris*, and others. We summarized the progress of metabolic engineering of xanthophyll according to four categories: hydroxy-, keto-, epoxy-, and epoxy-xanthophyll derivatives. Furthermore, we highlighted strategies to increase xanthophyll production in engineering microorganisms from the aspects of the protein engineering of key enzymes, compartmentalization of metabolic pathways in organelles, enhancement of metabolic flux, network regulation of metabolic pathway, and the engineering host selection. Finally, we also extracted the common and critical issues that hinder its heterologous production and proposed some promising suggestions to break through the bottleneck mentioned above. This review aims to present relevant researchers with a holistic understanding of the current development of xanthophyll metabolic engineering and to provide a global perspective for subsequent studies.

## 2. Heterologous Synthesis of Xanthophyll in Model Microorganisms

In this section, we present the research progress of different types of xanthophyll that have been synthesized by metabolic engineering in various model microorganisms, including the basic ideas and general metabolic engineering procedures, as well as some key issues that limit the production of xanthophyll. Table 1 lists all engineering microbial species of xanthophyll synthesis that have the highest yields or titers to date.

### 2.1. General Strategy for the Xanthophyll Production by Metabolic Engineering of Model Microorganisms

Briefly, the microbial metabolic engineering of xanthophyll involves the docking of the exogenous pathway of xanthophyll synthesis with the endogenous core metabolism of the microbial host, allowing the flows of carbon and energy to the synthesis of xanthophyll.

In model microorganisms, glucose or other carbon sources undergo glycolysis to produce acetyl coenzyme A (acetyl-CoA) and pyrurate. The acetyl-CoA and pyrurate then pass through the ergosterol synthesis pathway of the host cell, MVA (in plants, yeast, and algae) or MEP (in plants and bacteria) [16,17], to produce geranylgeranyl diphosphate (GGPP), an intermediate metabolite of the ergosterol pathway. GGPP is the connect point between core microbial metabolism and exogenous carotinoid synthesis pathways [45]. The subsequent synthesis of carotene and xanthophyll requires various heterologous carotenogenic enzymes and cofactors to convert GGPP further [18,19,20].

Experimental manipulation is used to introduce the genes of carotegenesis enzymes and cofactors into the microorganism and utilize the transcriptional, translational, and metabolic regulatory functions of the microbial host to control the expression of these genes. The key genetic elements involved in this process include highly active key enzyme genes, cofactor genes matching the key enzymes, various promoters and terminators, etc. The key enzyme genes and cofactor genes will be described in detail in Section 2.2., and the structures of expression cassettes consisting of the most effective promoters, genes, and terminators are listed in Table 1.

### 2.2. Progress in Metabolic Engineering Synthesis of Various Xanthophylls

#### 2.2.1. Hydroxy-Xanthophyll

Hydroxy-xanthophyll mainly refers to zeaxanthin, lutein, β-cryptoxanthin, zeinoxanthin, etc. The β-cryptoxanthin and zeinoxanthin are the intermediate metabolites of zeaxanthin and lutein [9,21,23], respectively, and the metabolic engineering of model microorganisms taking these intermediate metabolites as the end products has not yet been reported, so this section focuses on the engineering biosynthesis of zeaxanthin and lutein.

Zeaxanthin is the first xanthophyll in the β-carotene branch of the xanthophyll synthetic pathway and is the precursor of various high-value xanthophyll, such as astaxanthin, violaxanthin etc. (Figure 1). Unfortunately, although the production of zeaxanthin in various microbial engineering hosts has shown an increasing trend in recent years, it is still too low, with a maximum yield of only 18.7 mg/g [46], and it is the speed-limiting step of the engineering synthesis of downstream xanthophyll.

The reported model microorganisms for zeaxanthin synthesis include *E. coli*, *S. cerevisiae*, and *Y. lipolytica*. Zeaxanthin engineering synthesis is generally achieved by first introducing the exogenous β-carotene pathway into the host to construct a carotene chassis strain; the heterologous CrtZ is then expressed in this chassis strain to hydroxylate the β-ring at each end of β-carotene to produce zeaxanthin. The CrtZ gene has been cloned from a variety of algae and bacteria and applied to the metabolic engineering synthesis of zeaxanthin. In *E. coli*, compared with those from *Pantoea agglomerans* and *Haematococcus pluvialis*, the CrtZ of *Pantoea ananatis* has the highest β-carotene hydroxylation activity [46,47,48,49]. In *Y. lipolytica*, the catalytic activity of CrtZ from *Brevundimonas Vesicularis*, *H. lacustris* and *P*. *ananatis* was compared [50], and *P*. *ananatis* CrtZ had the highest activity, as in *E. coli*. In *S. cerevisiae*, the synthesis of zeaxanthin was mainly used as a reporting approach to study the feasibility of experimental methods such as multi gene co-transformation [51,52]; research aimed at improving the yield of zeaxanthin is rare. The discovery, optimization, and heterologous functional expression of highly active CrtZ are critical and urgent problems for zeaxanthin synthesis.

In addition to the above CrtZs, CrtZ proteins sequences of other species obtained by gene prediction or protein sequence alignment can be found on the NCBI GenBank database published in recent years, such as *Massilia varians* (GenBank: BDT58294.1), *Xanthomonadaceae bacterium* (GenBank: RZA32178.1), *Oxalobacteraceae bacterium* (GenBank: USX26554.1), etc. Their identities of protein sequences aligning against the CrtZ of *P. ananatis* are generally in the range of 60–80%, which is not lower than the identities between the above reported CrtZs. The potential of these putative CrtZ applications for the metabolic engineering of zeaxanthin also deserves further exploration.

Another important representative of hydroxyl-xanthophyll is lutein. The metabolic engineering of lutein is more complex and difficult than zeaxanthin due to the asymmetric cyclization of lycopene. α- carotene is the direct precursor of lutein, and the β-ring and ε-ring on the two ends of α- carotene are catalyzed by lycopene β-cyclase and ε-cyclase, respectively (Figure 1). The hydroxylation of the β-ring and ε-ring are catalyzed by two cytochrome P450 enzymes, CYP97A and CYP97C, respectively. These hydroxylation reactions require ferredoxin-NADP+ reductase (FNR), with ferredoxin redox partner, NADPH, and flavin adenine dinucleotide (FAD) as cofactors [53,54,55].

Studies have shown that the heterologously expressed activity of β-cyclase is higher than that of ε-cyclase, leading to a preference for lycopene for the formation of β-carotene with two β-rings [56,57,58]. Therefore, in addition to the hydroxylation, the asymmetric cyclization of lycopene is also a hindrance to lutein production in model microorganisms. The reported engineering microorganisms producing lutein are only *E. coli* and *S. cerevisiae*, and the yield is very low. In *E. coli*, through the screening of ε-cyclase from three sources (*Lactuca sativa*, *Tagetes erecta*, and *Marchantia polymorpha*) and CYP97C from nine sources (*Chlamydomonas reinhardtii*, *H. pluvialis*, *Brassica napus*, *Chenopodium quinoa*, *Oryza sativa*, *L. sativa*, *Nicotiana tabacum*, *Helianthus annuus*, and *M. polymorpha*), only the enzymes from *M. polymorpha* have lutein biosynthetic activity [56,59]. In *S. cerevisiae*, the synthesis of lutein can be achieved by the co-expression of β-cyclase from *Xanthophyllomyces dendrorhous*, ε-cyclase from *Tagetes erecta*, and CYP97A and CYP97C from *Arabidopsis thaliana* [57,58].

#### 2.2.2. Keto-Xanthophyll

Among all keto-xanthophylls (including astaxanthin, canthaxanthin, echinenone, etc.), astaxanthin has received the most attention due to its strongest antioxidant activity; this promotes the most in-depth research and the highest fermentation yield, and has shown great industrialization potential. Under controlled bioreactor fermentation, the maximum yield can reach tens of mg/g DCW [60], which could be comparable to a yield of approximately 40 mg/g DCW of the industrial algae natural-producer *H. pluvialis* [61].

The most commonly used model microorganisms for the production of astaxanthin include *E. coli*, *S. cerevisiae*, and *Y. lipolytica* [24,62]. *Kluyveromyces maximus* [63,64], *P. pastoris* [65,66], *Corynebacterium glutamicum* [67,68] etc., as hosts have also been reported.

The first step in astaxanthin synthesis by metabolic engineering is the heterologous functional expression of CrtW and CrtZ. Since both enzymes involve two substrates, there is an issue of substrate preference. Therefore, the yield of astaxanthin is closely related to the enzymatic activity, the substrate preference, and the combination of the two enzymes. Accordingly, the first step of almost all metabolic engineering research on the heterologous production of astaxanthin is the screening and adaptability analysis of these two enzymes. In *E. coli*, the combination of CrtW from *Brevundimonas* sp. SD212 and CrtZ from *Pantoea* sp. (including *P. agglomerans* and *P. ananatis*), or the CrtZ from *Paracoccus* sp. PC1 as a supplement to CrtZ from *Pantoea* sp., produces the highest astaxanthin yield [69,70,71,72,73,74,75]. The CrtZs from *Pantoea* sp. and *Paracoccus* sp. PC1 have different substrate preferences; a high conversion efficiency from β-carotene and canthaxanthin to astaxanthin can be achieved when the two enzymes have a combinatorial expression at a specific copy number ratio [69]. In *S. cerevisiae*, the higher astaxanthin production comes from the combination of the mutant BKT (H165R/V264D/F298Y/M1T/N188D/L271R) and CrtZ (L288R) of *H. pluvialis*, or the combination of the CrtW of *B. vesicularis* and CrtZ of *Agrobacterium aurantiacum* [76,77,78,79,80,81,82,83]. In *Y. lipolytica*, Wang [25] and Ma [84] et al. reported that the combination of the CrtZ from *Paracoccus* sp. and the CrtW from *H. pluvialis* yielded a higher astaxanthin production. Furthermore, the result of Wang et al. also showed that astaxanthin cannot be detected with the co-expression of CrtW and CrtZ from *H. pluvialis* in *Y. lipolytica*, On the contrary, Zhu [67] and Tramontin [85] reported that a higher astaxanthin production was produced by these CrtW and CrtZ from *H. pluvialis*. This contradiction needs to be further verified. In general, no matter the type of host, the bacteria CrtW and CrtZ usually have higher activity, followed by algae enzymes, and *X. dendrorhous* and plants (such as *Adonis aestivalis*) have very low or almost no activity [82,86].

Almost all reports indicate that CrtZ is a rate-limiting enzyme when the astaxanthin synthetic pathway is introduced in a model microbial host, which means the conversion from β-carotene to zeaxanthin, or from canthaxanthin and echinenone to astaxanthin, which is a key step for limiting the production of astaxanthin. Thus, in addition to the CrtZs listed in Table 1, the functional identification of other putative CrtZs mentioned in 2.1 may also be of significance for the synthesis of astaxanthin.

Canthaxanthin is another keto-xanthophyll that is widely used commercially. At present, only *E. coli* [87] and *S. cerevisiae* [88] have been reported as engineering microorganisms that take canthaxanthin as the target product. Theoretically, the synthesis of canthaxanthin does not require CrtZ, and its production should be higher than astaxanthin. Indeed, comparing the experimental results of Ye et al. [80,88], the yield of canthaxanthin (approximately 10–15 mg/g) in *S. cerevisiae* was higher than that of axtathophyll (5.7 mg/g) when almost the same strategies and shake flask culture conditions were used. This further demonstrates the rate-limiting effect of CrtZ.

#### 2.2.3. Epoxy-Xanthophyll

Epoxy-xanthophyll mainly includes violaxanthin and antheraxanthin. Antheraxhanthin was mentioned mainly as the intermediate metabolite of violaxanthin, so we only focused on the engfigureineering biosynthesis of violaxanthin in this section. The biosynthesis of violaxanthin by metabolic engineering is achieved by prolonging the pathway of zeaxanthin with the introduction of ZEP into a zeaxanthin-producing microbial host. Although the cloning and heterologous expression of ZEP have been studied for nearly 30 years [89,90,91,92], as far as the metabolic engineering of violaxanthin is concerned, it has only been described in detail in recent years.

ZEP belongs to FAD-dependent monooxygenase and catalyzes the epoxidation of zeaxanthin in the presence of NADPH, FAD, ferredoxin (FD), and ferredoxin-NADPH oxidoreductase (FNR) [93,94]. Therefore, the synthesis of violaxanthin in an engineering host requires the reducing power and the redox pair ether from the endogenous metabolism of the host or the functional heterologous expression of relative genes. In general, the reducing power of host cells cannot meet the needs of an efficient synthesis of violaxanthin, and not all redox pairs can transfer the electron to ZEP due to the specificity of ZEP for the redox partner. Therefore, the first challenge of violaxanthin metabolic engineering is the heterologous expression of highly active ZEP and its matching redox partner. The reported microorganism hosts for violaxanthin synthesis include *E. coli* and *S. cerevisiae*. In *E. coli*, the activity of ZEPs from seven higher-plants (*Capsicum annuum*, *A. thaliana*, etc.), one liverwort (*M. polymorpha*), and one algae (*Phaeodactylum tricornutum*) was compared [95]. The *C. annuum* ZEP had the highest activity, whereas that of *Prunus ameniaca*, *Zea mays*, *M. polymorpha*, and *P. tricornutum* showed extremely low activity in *E. coli*. Although the NADPH and electron transport system of *E. coli* can make ZEP active, the yield of violaxanthin is low. Exogenous redox partners, spinach ferredoxin and ferredoxin oxidoreductase, can improve the ZEP activity, whereas *Nostoc* sp. severely decreases its activity. The *Bacillus subtilis* glucose dehydrogenase as the NADPH-regenerating enzyme can increase the synthesis of violaxanthin in *E. coli*. Furthermore, the *E. coli* strains and ribosome-binding site (RBS) sequences also impacted the yield of violaxanthin. The best *E. coli* strain, JM101, had a yield of 231 μg/g of violaxanthin; however, the ratio of violaxanthin to total carotenoids was only 1.4–21%, which was still low. In *S. cerevisiae*, compared with the ZEPs of *A. thaliana* and *Solanum lycopersicum*, the *Haematococcus lacustris* ZEP showed the highest activity [96]. The redox partner from *A. thaliana* can improve the violaxanthin yield, whereas the yeast mitochondrial ferredoxin-like protein and its reductase has no effect on the yield. In the best violaxanthin-producing *S. cerevisiae*, the final yield of violaxanthin reached 7.3mg/g, corresponding to 58.4% of total carotenoids.

From the above, in *E. coli* and in *S. cerevisiae*, a considerable amount of upstream carotene remained, indicating that the catalytic efficiency of the heterologously expressed ZEP was low. The characterization and screening of ZEP and the matching redox partner from different species in various microbial hosts need to be further explored.

#### 2.2.4. Epoxy-Xanthophyll Derivative

Epoxy-xanthophyll derivatives include capsanthin, capsorubin, neoxanthin, fucoxanthin, diadinoxanthin, etc. Capsanthin, capsorubin, and neoxanthin are usually considered as the last metabolites of the carotenoid synthesis pathway in higher plants. The epoxy-xanthophyll derivatives have more complex terminal groups, and they may have multiple terminal groups, such as hydroxyl, ketone, epoxy, allenic, cyclopentane, cyclohexane, etc. (Figure 1).

Except for neoxanthin synthase (NSY) and the capsanthin/capsorubin synthase (CCS), the synthases for other epoxy-xanthophyll derivatives have not yet been identified or characterized. The NSY catalyze the opening of cyclohexenyl 5–6 epoxides at one end of violaxanthin to form an allenic group through a transient carbocation [31]. Although the NSY from *Lycopersicum esculentum* [31], *S. tuberosum* [97], *Arabidopsis* [32], and *Chinese Kale* [33] has been cloned, and the activity of *L. esculentum* NSY [98] conversing from violaxanthin to neoxanthin has been verified in *E. coli* (the substrate violaxanthin was added to the system and the yield of neoxhanthin was not mentioned), to date, neoxanthin production by microbial metabolic engineering has not been reported in detail.

**Table 1:** Non-native xanthophyll-producing microorganisms with the highest yield or titer ^a^.

Xanthophyll	Engineering Microbial Hosts	Key Enzymes	Natural Origin Species	Key Expression Cassettes ^b^	Methods or Principles of Host Transformation	Key Strategies	Yield (mg/g DCW)	Titer (mg/L)	Ref.
lutein	*E. coli*	ε-LCY	*M. polymorpha*	P_tac_-*IDI-CrtE-CrtB-CrtI-MpLCYb-MpLCYe-CrtZ-*T_rrnB_ and P_T7_-*MpCYP97C*-T_T7_- P_T7_-*MpLCYe*- T_T7_	Electroporation	Selection of ε-LCY and CYP97C from different species, decreasing the activity of β-LCY and increasing the copy number of ε-LCY gene		2.6	[56]
		β-LCY	*M. polymorpha*						
		CYP97C	*M. polymorpha*						
		CrtZ	*P. ananatis*						
	*S. cerevisiae*	ε-LCY	*A. thaliana*	P_TER1_-*tHMG1*-T_CYC1_, P_PGK1_-*CrtE03M*-T_ADH1_, P_PGK1_-*CrtYB11M*-T_ADH1_, P_TEF_-*CrtI*-T_CYC1_, P_ACT1_-*Gal4M9*-T_ADH1_, P_GAL1_-*CrtYB*-T_CYC1_, T_CYC1_-*CYP97A3*-P_GAL1_-P_GAL10_-*LUT1*-T_ADH1_-T_PGK1_-*FD3*-P_GAL2_-P_GAL7_-*RFNR1*-T_TPS1_, P_TEF1_-*PM^SeV-C^-At-LCYE*-T_CYC1_, P_GAL1_-*CYP97A3*-T_CYC1_	Chemical transformation	Selection of ε-LCY from different species, regulation of ratios of CYP97A3 and RFNR1/FD3, and hierarchical dynamic regulation based on the temperature-responsive promoter	4.53	19.92	[58]
		CrtYB	*X. dendrorhous*						
		CYP97A3	*A. thaliana*						
		Lut1	*A. thaliana*						
		RFNR1	*A. thaliana*						
		FD3	*A.s thaliana*						
zeaxanthin	*E. coli*	CrtZ	*P. ananatis*	P_T5_-*CrtEIBipi*-TTR, P37-*CrtY- 2CrtZ*-T_rrnB_, pZSP_IA44_-*MevT_TIGR_*-*MevB_TIGR_ IS*-2	Electroporation	Introduction and dynamic control of the MVA pathway of *S. cerevisiae* to increase the precursors supply and prevent the accumulation of toxic metabolites	18.7	58.05	[46]
	*S. cerevisiae*	CrtZ	*P. ananatis*	P_PDC1_-*CrtE*-T_PDC1_, P_TPI1_-*CrtB*-T_TPI1_, P_GPM1_-*CrtI*-T_GPM1_, P_GPD_-*CrtY*-T_GPD_, P_FBA1_-*CrtZ*-T_FBA1_	Chemical transformation	Zeaxanthin as a reporter gene for identification of promoter strength		0.74	[51]
	*Y. Lipolytica*	CrtZ	*P. ananatis*	P_TEF1N_-*CrtE*-T_xpr2_, P_TEF1N_-*CrtB*-T_xpr2_, P_TEF1N_-*CrtI*-T_xpr2_, P_TEF1N_-*CarRP*-T_xpr2_, P_TEF1N_-*CrtZ*-T_xpr2_	Frozen-EZ Yeast Transformation II Kit	High-copy-number integration of CrtZ gene into ribosomal DNA region		21.98 in YPD medium (3.2 in YNB medium)	[50]
astaxanthin	*E. coli*	CrtZ	*P. ananatis*	P_TM2_-*CrtEBIA*, P_T7_-*RLZ*-*CrtZ*-*RLW*-*CrtW*		Screening and regulation of promoters and RBSs	15.1	62	[75]
		CrtW	*Brevundimonas* sp. SD212						
	*S. cerevisiae*	CrtZ	*A. aurantiacum*	A high β-carotene producing strain with P_FBA1_-*CrtZ*-T_ADH1_, P_TDH3_-*CrtW*-T_TDH2_	Homologous recombination	Selection and optimization of combinations of CrtW and CrtZ from different species	6.05		[76]
		CrtW	*Alcaligenes* sp.						
	*Y. Lipolytica*	CrtZ	*H. pluvialis*	P_TEF_-*carRP*-T_XPR2,_ P_TEF_-*thmgR*-T_XPR2,_ P_TEF_-*GGS1*-T_XPR2,_ P_TEF_-*carB*-T_XPR2,_ P_TEF_-*CrtW*_-linker-RIDD_-T_XPR2_-P_TER_-*CrtZ*_-linker-RIAD_-T_XPR2_	Chemical transformation	Selection of CrtW and CrtZ from different species and fine-tuning their transcription	17.5		[60]
		BKT	*H. pluvialis*						
	*P. pastoris*	CrtZ		P_AOX1_-*CrtI*-T_CYC1_, P_AOX1_-*CrtE*- *CrtZ*-T_CYC1_, P_AOX1_-*CrtYB*-*CrtW*-T_CYC1_	CRISPR/Cas9	Astaxanthin as a reporter gene for marker-less integration of multigene pathways into *Pichia pastoris* via CRISPR/Cas9		Approximately 2.5	[66]
		CrtW							
	*K. marxianus*	CrtZ	*H. pluvialis*	P_KlLac4_-*CrtZ*-T_KlLac4_, P_ScGapDH_-*CrtE*-T_ScGap_, P_ScPGK_-*CrtZ*-T_ScPGK_, P_KlGapDH_-*kanMX*-T_ScGap_, P_ICL_-*CrtI*-T_35S_, P_KlPGK_-*BKT*-T_ScPGK_, P_KlADH1_-*CrtYB*-T_ScGap_, P_ScADH1_-*tHMG*-T_ScADH1_	Homologous recombination	Increasing the copy number of Hpchyb and BKT genes and modifying the Hpchyb by site-directed mutagenesis	3.125 in YPL medium, 5.701 in YPG medium		[64]
		BKT	*C. reinhardtii*						
	*C. glutamicum*	CrtZ	*F. pelagi*	P_tuf_-*CrtZ*-linker-*CrtW*	electroporation with xenogeneic plasmid DNA	Fusion expression of CrtZ and CrtW, increasing the expression of upstream enzymes, mediated medium composition	3.1		[68]
		CrtW	*B. aurantiaca*						
canthaxanthin	*E. coli*	BKT	*Anabaena variabilis*	P_Trc_-*CrtW*	Electroporation	Overexpression of host genes increases the carbon flux into the canthaxanthin biosynthetic pathway	Approximately 10.65	24.84	[87]
	*S. cerevisiae*	OBKTM29 (mutant BKT)	*H. pluvialis*	P_GAL1_-*mBKT*-T_CYC1_-P_GAL10_-*CrtE03*-T_ADH2_, T_CYC1_-*PM^SeV-C^*-*mBKT*-P_GAL1_-P_GAL10_-*CrtYB*-T_ADH2_, T_CYC1_-*PDR3*-P_GAL1_-P_GAL10_-*CrtYB*-T_ADH2_	Homologous recombination and CRISPR/cas9	Subcellular re-localization of OBKTM29 and its copy number adjustment both in the cytoplasm and on the periplasmic membrane, pleiotropic drug resistance (PDR) regulator overexpression	approximately 20–30	168	[88]
violaxanthin	*E. coli*	CrtZ	*P. ananatis*	P_lac_-*CrtE-CrtY-CrtI-CrtB-CrtZ*, P_lac_-*ZEP*, P_T7_-*gdh*		Selection of ZEP from different species and optimization of *E. coli* strain, expression vector, and ribosome-binding site (RBS) sequence	0.231		[95]
		ZEP	*C. annuum*						
		glucose dehydrogenase (gdh)	*B. subtilis*						
	*S. cerevisiae*	CrtZ	*P. ananatis*	P_TDH3_-*CrtYB*-T_CYC1_, P_TDH3_-*CrtI*-T_CYC1_, P_TDH3_-*CrtE*-T_CYC1_, P_TEF1_*-CrtZ*-linker-*trZEP*-T_ADH1_, P_TEF1_-*trRFNR1*-T_ADH1_, P_PGK1_-*trFD3*-T_CYC1_	Modified homologous recombination	Selection of CrtZ, ZEP and redox partner from different species and their truncated variants, increasing gene copy number of upstream carotenogenic genes	7.3		[96]
		ZEP	*H. lacustris*						
		RFNR1	*A. thaliana*						
		FD3	*A. thaliana*						
capsanthin	*E. coli*	CrtZ	*P. ananatis*	P_lac_-*HpIDI*-*CrtE*-*CrtY*-*CrtI*-*CrtB-CrtZ*, P_tac/T7_-*CCS_M40_*-*CaZEP*		A particularly high expression of CCS		0.5	[97]
		ZEP	*C. annuum*						
		CCS	*C. annuum*						

^a^ Under shake flask culture conditions. Under the conditions of fermentation tank, it is difficult to compare the yield or titer due to different fermentation types, medium, and fermenting duration. In order to relatively accurately reflect the ability of engineered cells to synthesize xanthophyll, the highest yields or titers here are for shake flask culture conditions. ^b^ Capital P and T indicate promoters and terminators, respectively.

Capsanthin is the only epoxy-xanthophyll derivative whose synthesis has been achieved by microbial metabolic engineering. Capsanthin and capsorubin are synthesized by CCS catalysis from antheraxanthin and violaxanthin, respectively (Figure 1). Although there are at least ten protein sequences from different species identified as CCSs in NCBI GenBank, only CCSs from *Capsicum annuum* [99] and *Tiger lily* [30] have been cloned and verified for their functions so far.

CCS is a multifunctional enzyme with capsanthin/capsorubin synthesis and β-carotene synthesis, and its activity requires the simultaneous existence of FAD and NADPH. In addition, it was also found that CCS catalyzes the reverse reaction in the presence of NAD^+^; that is, the reverse conversion from capsorubin to violaxanthin [29,100,101,102]. In 2021, by introducing ZEP and CCS from *C. annuum* into β-carotene-producing *E. coli*, combined with greatly enhancing the expression of CCS, the production of capsanthin was realized with a titer of 0.5 mg/L [103]. The high-level expression of CCS is necessary for the production of capsanthin; otherwise, only lycopene cyclization activity can be detected. Moreover, in the best strains, there are still considerable residues of precursors (zeaxanthin, antheraxanthin, and violaxanthin), which proves that CCS as well as ZEP are the rate-limiting enzymes of the capsanthin synthetic pathway in the engineering of *E. coli*. In addition, no capsorubin was detected in this study, which was consistent with the phenotype of the capsanthin accounting for a large amount in pepper, whereas the capsorubin was only a minor product [37]. Therefore, it can be speculated that CCS may have a preference for violaxanthin over antheraxanthin. Any metabolic engineering of capsanthin or capsorubin in yeast has not yet been reported.

From the above, it is known that the metabolic engineering of epoxy-xanthophyll derivatives is still at the beginning stage. The cloning and characterization of key enzymes, substrate preference, and cofactor specificity need to be further investigated. From the catalytic properties of CCS, it can be speculated that the key enzymes, such as the NSY, fucoxanthin, or diadinoxanthin synthetic enzymes, may also involve multiple substrates or multiple products, which implies that the metabolic engineering of epoxy-xanthophyll derivatives is more difficult to control.

## 3. Metabolic Engineering Strategies of Model Microorganisms for Xanthophyll Synthesis

**Figure 3:**
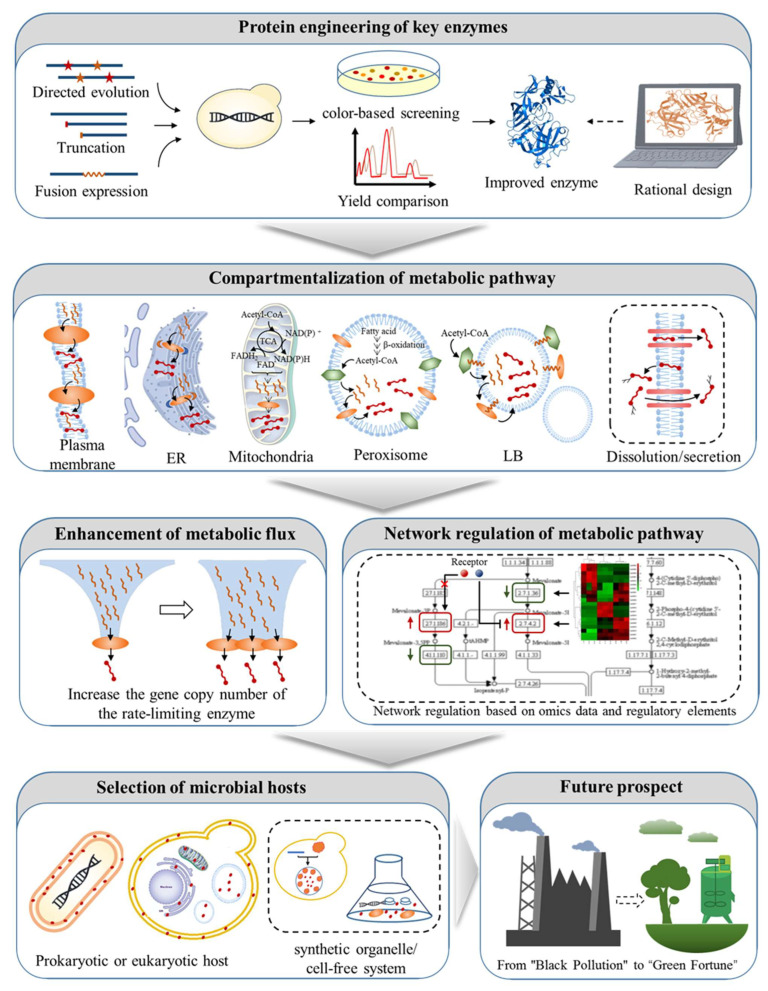
Metabolic engineering strategies for xanthophyll synthesis in model microorganisms illustrating five levels: protein engineering of key enzymes, compartmentalization of metabolic pathway, enhancement of metabolic flux, network regulation of metabolic pathway, and selection of microbial hosts. Protein engineering of key enzymes: directed evolution, truncation, or fusion expression is used to manipulate the enzyme protein through the color-based screening or yield comparison, obtaining an enzyme with improved catalytic activity. Dashed box: rational design of enzymes based on three-dimensional structure of proteins and computer modeling for rapid acquisition of improved enzymes. Compartmentalization of metabolic pathway: localization of metabolic pathways to various organelles, exploiting the different characteristics of various organelles to improve reaction efficiency, increase storage capacity, and reduce cytotoxicity. Dashed box: glycosylation of xanthophyll or engineering transport to promote xanthophyll solubilization or secretion, breaking through various regulatory barriers of the host. Enhancement of metabolic flux: breaking the metabolic flux bottleneck by increasing the gene copy number of the rate-limiting enzyme. Network regulation of metabolic pathway (in dashed box): based on omics data and regulatory elements, regulating the metabolic network at a holistic level to adapt host cell to the xanthophyll synthetic pathway. Selection of microbial hosts: microbial hosts are selected according to different research purposes (e.g., characterization of enzyme function, validation of metabolic pathway availability and regulatory circuits, high yield, etc.). Dashed box: custom synthesized organelles specifically for xanthophyll synthesis in microbial host, and in vitro synthesis of xanthophyll using a cell-free system. Future prospect: upgrade of industrial production model from “Black Pollution” to “Green Fortune”. The dotted line indicates future research directions for the new strategies.

After describing the overall idea and production status of xanthophyll synthesis by metabolic engineering model microorganisms, in this section, we will present comprehensive metabolic engineering strategies to improve xanthophyll production. For a certain xanthophyll synthesis, multiple strategies are usually used in combination. Since these strategies can be used universally and considered to make related works more convenient to draw on, we will present them in categories from five levels: the protein engineering of key enzymes, compartmentalization of metabolic pathway, enhancement of metabolic flux, network regulation of metabolic pathway, and microbial host selection (Figure 3).

### 3.1. Protein Engineering of Key Enzymes

#### 3.1.1. Directed Evolution of Key Enzymes

Among the key enzymes (referring to the enzymes located downstream of lycopene or β-carotene in the xanthophyll synthetic pathway and directly synthesizing xanthophyll), the ε-LCY from Tagetes erecta, CrtZ from *H. pluvialis* and *B. vesicularis*, BKT/CrtW from *H. pluvialis*, *Brevundimonas* sp. SD212, *Sphingomonas melonis*, and *Rhodocuccus erythropolis* have been directed to evolve for functional improvement (Table 2). Directed evolution was achieved mainly by the combination of random mutation and high-throughput screening. Error-prone PCR and a color-based screening system are the most commonly used methods for random mutation and high-throughput screening, respectively. The colony color of a random mutant library established by error-prone PCR is changed with the accumulation of the target product.

In the conversion from lycopene to δ–carotene catalyzed by the ε-LCY, the corresponding colony color was changed from red to yellow or orange [57]. The CrtW/BKT converted β-carotene to keto-carotene, and the color changed from yellow to red [81,104]. In an astaxanthin-producing host, the mutation of CrtZ can be combined with BKT, resulting in the β-carotene changing to astaxanthin and the colony color changing from yellow to red [80]. However, for CrtZ, it converts β-carotene to zeaxanthin, and the change in colony color is not obvious. There are not yet any reports on the mutation of CrtZ alone. The catalytic activity of the mutant enzyme screened from error-prone PCR may be further improved by saturation mutagenesis. For example, the catalytic activity of the F61S, a mutant ε-LCY, was increased by 67% compared with the wild type, a further elevation of 32% was reached by saturation mutagenesis, which was performed for the substitution by all other amino acids at site 61, and the highest activity mutant, F61N, was identified [57]. In addition to error-prone PCR, DNA shuffling and the staged extension process (StEP) [105,106] are also effective random mutation methods, especially for the enzymes in one family, such as LCY family (including LCY, CCS, NSY), or homologous enzymes from different species (such as the CrtZs from *Pantoea* sp. and *Paracoccus* sp.). Their amino acid sequence is highly consistent, but their catalytic activities and substrate specificity are different.

In addition, the color-based screening system is not applicable to enzymes that do not cause colony color changes, such as CrtZ and ZEP, so other high-throughput screening methods need to be established. It is reported that xanthophylls have a stronger resistance to hydrogen peroxide than carotene [77,107]. Therefore, a screening based on hydrogen peroxide concentration may also be another potential option. Besides random mutation, site-directed mutation is more suitable for the functional identification of specific domains. For instance, the fatal effect of site 295E in the 293-FLEET-297 motif of CCS on the catalytic activity was proved by the site-directed mutation [101]. Other commonly used enzymes for xanthophyll production, such as CrtZ from *Pantoea* sp. and *Paracoccus* sp., ZEP and CCS from pepper, ε-LCY and α- carotene hydroxylase from *A. thaliana*, etc., have not been reported in directed evolution.

In addition to catalytic activity, substrate specificity is also an important aspect of directed evolution. In a lycopene-producing *S. cerevisiae*, through the directed evolution of a bifunctional enzyme CrtYB, its lycopene β-cyclase activity was eliminated, but the phytoene synthase activity was retained and the lycopene yield was correspondingly increased [108]. Similar studies focused on the changing substrate specificity of xanthophyll synthesis enzymes have not yet been reported.

#### 3.1.2. Truncation of Transit Peptides

In plants and algae, the key enzymes of xanthophyll synthesis are targeted at the plastid, such as chloroplast, chromoplast, etc. [109,110]. There is usually a transit peptide located in the N-terminal of the key enzymes that introduces these enzymes into the plastid and is cleaved by proteases after import. Truncation makes the mature enzymes exploit complete catalytic activity. In metabolic engineering microbes, the truncation of the transit peptide can increase the catalytic activity of heterologously expressed key enzymes in most cases. Table 3 lists the truncated enzymes and their truncated positions that were proven to have a functional improvement.

The length of the transit peptide is usually determined by prediction (e.g., ChloroP1.1 server, and others), and the real truncation site of the transport peptide in natural organisms is not accurately known. Therefore, the enzymatic activity can be optimized by testing different truncated sites around the predicted site. For example, when the ZEP variants from *H. lacustris* with different lengths of N-terminal truncation were expressed in *S. cerevisiae*, the variants with a truncation of 30 and 59 amino acids had a higher activity compared with the truncation of 80 amino acids. The catalytic activity was abolished when the truncated length reached 100 residues [96]. A truncation of at least 25–30 residues was required for the catalytic activity of ZEPs from *A. thaliana* and *S. lycopersicum*, and the full-length enzymes were inactive in *S. cerevisiae* [96].

In addition to the key enzymes, the redox partner proteins may also contain the transit peptide for transport to certain organelles. The N-terminal truncation of the redox partner protein in a heterologous microbial host may increase their capacity of transfer electrons. For example, when ferredoxin-NADPH oxidoreductase 1 (RFNR1) and ferredoxin 3 (FD3) from *A. thaliana* as redox partners of ZEP are used in violaxanthin-producing *S. cerevisiae*, the truncation of the redox partner (RFNR1 truncated at the 65th residue and FD3 truncated at the 49th residue) increased the violaxanthin accumulation 2.2-fold [96].

There are also other situations. In *E. coli*, the catalytic activity of full-length ZEP from *C. annuum* was higher than the truncation variant of 56 residues of the N-terminal; this truncated position was chosen according to the amino acids sequence aligned with the ZEP from *A. thaliana*. In addition, the full length or truncation for CCS from *C. annuum* has no significant effect on its capsanthin/capsorubin synthesis activity [103].

#### 3.1.3. Fusion Expression of Key Enzymes

The synthesis of xanthophyll in a microbial host is usually involved in the continuous catalytic reaction of multiple enzymes, so the efficiency of the interaction between the metabolic intermediate and enzyme is a factor affecting the xanthophyll production. In addition, xanthophyll and its synthetic enzymes are mainly present in membrane localization; thus, its lateral diffusion rate in the membrane may also affect the catalytic efficiency of the enzymes. It has been found that the fusion expression of two adjacent enzymes in the synthesis pathway can improve the production of xanthophyll. This might be achieved by shortening the distance between the enzyme and the substrate or increasing the concentration of the substrate in vicinity of the enzyme, which improves the utilization of intermediate metabolites.

In order to simultaneously exert the activity of the two fused enzymes, the method of fusion, including the relative position of the two enzyme proteins and the rigidity/flexibility or length of the linker, all need to be considered. Cataldo et al. [96] evaluated the effect of the fusion expression of CrtZ from *P. ananatis* and ZEP from *H. lacustris* on the yield of violaxanthin. They found that the CrtZ attaching as the N-terminal module was necessary for maintaining the two enzyme activities, and the rigid linker was better than the flexible one. Compared with the individual expression of the two enzymes, the fusion construct can more efficiently channel zeaxanthin toward violaxanthin in the engineering of *S. cerevisiae*. Nogueira et al. [70] analyzed the effect of the length of the flexible linker and relative arrangement of CrtZ and CrtW, which were from the *Brevundimonas* sp. strain SD212, on the activity of the fusion enzyme. The results indicate that the level of the target product astaxanthin in engineering *E. coli* was increased 1.4-fold at the fusion condition and that the size of linkers had no significant effect. From the above-mentioned fusion methods, it seems that the CrtZs from bacteria must be located at the N-terminal of the fusion construct.

Ma et al. [84] combined the CrtZ with CrtW (from *H. pluvialis* and bacteria *Paracoccus* sp., respectively) through a flexible linker and found that the fusion enzyme could improve the production of astaxanthin in *Y. lipolytica* and that the CrtW-linker-CrtZ had a higher activity than the CrtZ-linker-CrtW. In contrast, Zhu et al. [60] reported that the combinations of CrtW and CrtZ (both enzymes are from *H. pluvialis*), whether through a flexible linker or RIAD/RIDD interaction peptides, decrease the yield of astaxanthin in *Y. lipolytica*. The above two reports implied that the CrtZ from the alga *H. pluvialis* did not have to be located at the N-terminal of a fusion construct. The connection mode of the two enzymes in the fusion enzyme needs to be evaluated experimentally to obtain the maximum activity.

### 3.2. Compartmentalization of Metabolic Pathway

In natural species, organelles, such as chloroplasts and chromoplasts, compartmentalize the metabolic pathways of xanthophyll, ensure the efficient implementation of metabolic flux, and prevent the cytotoxicity of metabolites. When the xanthophyll synthetic pathway is introduced into the heterologous microbial host, these enzymes and metabolites tend to diffuse all over the intracellular membrane, and a small fraction may also be released into the cytoplasm. This diffusion reduces the possibility of contact between the enzyme and substrate, and the excessive accumulation of intermediates may affect the cell membrane fluidity [111,112].

In *E. coli*, which has no subcellular compartments, the membrane is the main targeted organelle for the xanthophyll synthetic pathway. An improvement in the load capacity of the membrane and membrane targeting of enzymes, which reduced the distance between the substrates and the enzymes, was demonstrated to increase xanthophyll production. For example, Lu et al. generated longer and larger cells through deleting the morphology/membrane-related genes (lpp and bamB), thereby increasing astaxanthin production in *E. coli* [113]. Park et al. localized the BKT to the membrane of *E. coli* using the signal peptides of OmpF and TrxA, and the yield of astaxanthin was increased 2.8-fold [114]. Ye et al. localized the CrtW and CrtZ to the membrane of *E. coli* using the GlpF protein, allowing for a 215.4% astaxanthin production increase [72]. In addition to *E. coli*, in yeast, membrane targeting was also proven to be beneficial to the synthesis metabolites. Bian et al. localized the ε-LCY to the plasma membrane of *S. cerevisiae* by PM^SeV-C^, and the α-carotene production was increased 2.2-fold [57].

Yeast cells contain a variety of organelles, including an endoplasmic reticulum (ER), Golgi apparatus, vacuole, mitochondria, peroxisomes, and lipid body. Each subcellular organelle has a unique physiochemical environment [115]. Many reports have shown that the subcellular localization of metabolic pathway could improve the terpenes production; therefore, yeast subcellular engineering for the synthesis of related compounds is emerging as a blooming field. At present, the ER, mitochondria, peroxisome, and lipid body of yeast can realize the artificial localization of heterologous enzymes. The locating signal peptides for various subcellular organelles are listed in Table 4.

The ER is the site of protein synthesis, folding, modification, and assembly with strict dynamic regulation [116,117]. The overexpression of exogenous proteins often leads to ER stress responses, such as the unfolded protein response (UPR) and ER-associated degradation (ERAD), which accelerate proper protein folding or guide protein degradation [118]. In the context of ER locating, the correct folding of the enzyme protein and the loading capacity of ER have an important effect on xanthophyll production. Ma et al. [84] localized the CrtZ-linker-CrtW fusion protein into the ER of *Y. lipolytica*, and the titer of astaxanthin was increased approximately two-fold compared with the fusion protein before localization. The change in ER morphology may also affect the production of xanthophyll. Although there is no relevant report on xanthophyll, the extension of ER has been proven to improve the production of upstream precursors in the xanthophyll pathway or other terpene chemicals. Kim et al. [119] expanded the ER space to enhance the protein synthesis and folding capacity of ER by overexpressing INO2, an ER-size-regulatory factor that causes a productive increase in squalene (a competitive substrate of carotenoids synthesis) in *S. cerevisiae*. The deletion of the phosphatidic acid phosphatase PAH1 could also have expanded the ER that stimulated the expression of targeted enzymes and ultimately increased the production of a triterpene and its derivative [120].

Mitochondria are the sites of tricarboxylic acid cycle (TCA) and energy synthesis. The initial precursor of the carotenoid pathway—the acetyl-CoA—and a wide range of cofactors, including NAD(P)H, NAD(P) ^+^ and FAD, are located in this organelle, supplying an abundance of precursors and cofactor pools for xanthophyll synthesis [115]. Yuan et al. [121] localized the FPP biosynthetic enzymes into the mitochondria and thereby increased the production of FPP and its derivative in *S. cerevisiae*. As the precursor of xanthophyll, the increase in FPP production may also benefit the synthesis of xanthophyll.

Peroxisome is the site of fatty acid β-oxidation surrounded by a phospholipid monolayer [122]. The endogenous acetyl-CoA pool is produced in the β-oxidation process. The peroxisome locating of the MVA pathway could increase the GPP synthesis, bypass the endogenous competition for GPP, and limit the cytotoxicity of the downstream metabolites. Cofactors such as ATP and NADPH can also be involved in intensive synthesis in the peroxisome by locating the relative enzymes [123,124]. In addition, the overexpression of oleate-dependent transcription factors Adr1, Oaf1, and Pip2 [123,125] or the simultaneous deletion of Pex30 and Pex31 [126] could expand the peroxisome size and improve the import capacity. This may be beneficial for the localization and loading of enzymes of xanthophyll synthesis. Notably, almost all of the enzymes of xanthophyll synthesis contain transmembrane regions, which correspond to the phospholipid bilayer. However, peroxisome is a monolayer [122], so after being localized to the peroxisome, whether the enzyme is located on the membrane or stored in the peroxisome remains to be determined. Another organelle, the lipid body, also faces this problem.

The lipid body (LB), also called lipid droplet (LD), is the site of the storage of triacylglycerols and steryl esters in yeast. The LB has a hydrophobic interior wrapped by the phospholipid monolayer [123]. All the carotenoids are hydrophobic; therefore, many studies have focused on LB localization for the synthesis of carotenoids, especially the oleaginous yeast *Y. lipolytica* [127], which contains high amounts of LB and acetyl-CoA compared with *P. pastoris* and *S. cerevisiae*, and has specific and potential advantages for xanthophyll synthesis. Studies show that lycopene and β-carotene are mainly localized on the cell membrane when they are produced in *S. cerevisiae*, and that membrane localization may lead to cytotoxicity [128]. However, when they are produced in *Y. lipolytica*, lycopene and β-carotene are mainly stored in the LB [129]. Moreover, genes (such as YlDga1 and YlDga2) that regulate the number and size of LBs in *Y. lipolytica* have been verified, providing the possibility of using the LB as a subcellular factory to improve xanthophyll production and the storage capacity [130]. Nevertheless, the research on LB targeting for xanthophyll synthesis is still inadequate, and the advantage of LBs has not been exploited. Ma et al. [84] found that the subcellular location (including the LB, mitochondria, and peroxisome) of the CrtW-linker-CrtZ fusion enzyme could increase the quantity of astaxanthin. Meanwhile, they also found that the peroxisome location achieves the highest production but the LB location is lower, which may be due to the fact that the LB has a lower carrying capacity for the pathway enzymes than the peroxisome.

In addition to what was mentioned above, reports on the subcellular localization of other xanthophyll synthetic pathways are still few. Compared with carotene, xanthophyll has its own physicochemical properties, such as a relatively higher molecular polarity, stronger antioxidant activity, many key enzymes that need the synergy of cofactors, and a longer pathway bringing more by-products, which leads to a higher loss of intermediates. These inherent characteristics of xanthophyll synthesis affect the selection of subcellular organelles. Therefore, research on the adaptability between xanthophyll and different subcellular organelles may be a growth point for increasing xanthophyll production.

### 3.3. Enhanced Metabolic Flux of Xanthophyll Synthetic Pathway

Xanthophyll synthesis in heterologous microbes is achieved through the channeling of a series of precursors into the xanthophyll metabolic pathway flow. The metabolic flux of pathway flow directly influences xanthophyll production. Along with the evolution of key enzymes and organelle location, strengthening this metabolic flux is an important strategy for increasing the xanthophyll production. In this section, the main ways to strengthen the xanthophyll metabolic flux will be elaborated.

Increasing the gene copy numbers of key enzymes, especially for the rate-limiting enzymes, to enhance the expression of key enzymes is the common way to strengthen the xanthophyll metabolic flux. For example, increasing the gene copy number of the rate-limiting enzyme, such as ε-LCY, CrtYB, ZEP, and CrtZ, can significantly increase the production of metabolites, whereas the non-rate-limiting enzyme, such as CrtE, BKT, and CrtW, has no effect [25,56,81,86,96]. The overexpression of heterologous enzymes may lead to cell stress and metabolic imbalance or reach the upper limit of the host expression capacity. There should be an optimal gene copy number corresponding to the highest xanthophyll production in a certain genetic background. Indeed, the optimal number of copies has been identified in several reports (Table 5). Notably, an extra CrtI copy can increase the β-carotene [131] or total carotenoid production in the β-carotene-producing *S. cerevisiae*, whereas it is lethal for the violaxanthin-producing *S. cerevisiae*. This phenomenon implies that *S. cerevisiae* is less tolerant to polar xanthophyll than hydrophobic carotenes [96]. Based on previous reports, increasing the copy number of key enzyme genes should be the first strategy. In addition to the enzymes mentioned above, the optimal copy number of other rate-limiting enzymes, such as ZEP, CCS, etc., has not been reported, which may be an efficient way to increase xanthophyll production.

In addition to the gene copy number of key enzymes, the cofactor supply is also a crucial factor for maintaining the xanthophyll metabolic flux. The cofactor is the competitive compound between the xanthophyll metabolic pathway and host metabolic network [132]. Not only does the whole xanthophyll metabolic pathway, from acetyl-CoA synthesis to the MVA/MEP pathway and finally to xanthophyll production, require the participation of cofactors, but so does the central carbon metabolism of the host, such as glycolysis, the TCA cycle, the pentose phosphate pathway (PPP), etc. Different xanthophyll synthetic enzymes need different cofactors.

The types of cofactors required for xanthophyll synthesis are described in Section 2.2. The metabolic demand for cofactors can be satisfied by strengthening the host’s own cofactor synthetic pathway or modifying the existing pathway to reduce the consumption of cofactors. The primary source of cytoplasmic NADPH is the oxidative PPP [133]. In *Y. lipolytica*, the astaxanthin production was increased by the overexpression of phosphorgluconate dehydrogenase, which is a key enzyme for NADPH synthesis in the PPP [25]. Meadows et al. [134] reported that both PDH-bypass and mevalonate synthesis reactions can be altered by the introduction of heterologous enzymes and reduce the consumption of ATP and NADPH, respectively. It seems that this strategy should also work for the synthesis of xanthophyll.

The synthesis and consumption of cofactors involves multiple major metabolic pathways and affects host energy and redox homeostasis. Designing and constructing energy conserving and more efficient pathways is a promising research direction for supporting a high flux of xanthophyll pathways while maintaining cell growth. Related strategies have been shown to be feasible in the microbial synthesis of other high-value compounds [132,134,135], but less research has been carried out in the xanthophyll case.

### 3.4. Network Regulation of Metabolic Pathway

The introduction of a heterologous pathway may unbalance the metabolic network of the host microorganism due to the deficit of the enzyme, cofactor, and energy. Meanwhile, heterologous metabolic pathways do not gain predominance and may lead to a waste of carbon and energy sources, which can also lead to a suboptimal productivity of the target metabolites.

The real-time dynamic regulation of the global metabolic network can maintain the host microorganism growth while adapting it to the efficient accumulation of target metabolites, as well as improving the host robustness during industrial fermentation [136]. According to available reports, studies regulating the xanthophyll metabolic pathway have mostly focused on the synthesis of intermediates in *E. coli* and *S. cerevisiae* [137,138,139,140]. Few studies have been conducted on the metabolic network regulation in other microbial hosts or for the end product synthesis of the xanthophyll pathway. Nevertheless, the available reports still provide a wealth of research ideas and biotechnological tools.

Multi-omics technologies (including genomics, proteomic, transcriptomics, metabolomics, etc.) offer the possibility of understanding the overall metabolic state of the host cell; the overexpression, low-expression, knockdown, and knockout can be used to regulate the expression level of enzymes; building new bridges between metabolites can optimize the inherent metabolic pathways of the host; constructing a dynamic regulatory circuit allows the host metabolic network to adapt to the metabolic flux of the target pathway in a timely manner. Shen et al. [46] analyzed the proteome of engineering *E. coli* in which the MVA pathway was dynamically regulated, and found that zeaxanthin overproduction in this host may be associated with the Embden–Meyerhof–Parnas (EMP) pathway, amino acid and fatty acid biosynthetic pathways, energy metabolism, and oxidative stress responses. Based on these results, they knocked out the oxidative-stress-related genes (*uspE* and *yggE*) to elevate reactive oxygen species (ROS) in *E. coli* cells, thereby stimulating the production of astaxanthin to cope with oxidative stress [113]. Similarly, the knockout of some other pressure response proteins can also improve the production of astaxanthin to varying degrees [141]. Through transcriptome analysis, Wang et al. [25] found that the mevalonate and ergosterol biosynthetic pathway, oxidative PPP, and antioxidant system were significantly down-regulated in the engineering of *Y. lipolytica* with astaxanthin overproduction. Although omics technologies can reveal the effects of the introduction of the xanthophyll pathway on host metabolic networks in a holistic manner, these effects are complex and comprehensive, and the improvement of xanthophyll production strains through metabolic network modulation based on omics data still remains a challenge. Nowadays, several metabolic network modulation strategies for regulating precursors of xanthophyll have been established.

In *E. coli*, a sensing and dynamic control system, including the sensor transcriptional factor catabolite repressor/activator (Cra), the signal (fructose-1, 6-diphosphate), and the control valve Cra-regulated promoter, was developed to regulate the glycolysis flux for mevalonate production [142]. Another control system consisting of the sensor NRI, the signal acetyl phosphate, and the control valve glnAp2 promoter was established to enhance lycopene production [143]. In *S. cerevisiae*, rewiring the central carbon metabolism by replacing the PDH-bypass with a non-native metabolic route enables the synthesis of acetyl-CoA while reducing the energy requirements and carbon losses and improving the redox balance [134]. Reducing the half-life of ERG20 (a kind of farnesyl pyrophosphate synthase in *S. cerevisiae*, converting GPP to FPP) in combination with the use of sterol-sensitive promoters to regulate the flux of the MVA pathway can promote the accumulation of GPP while maintaining sterols at the lowest level, which ensures cell growth. Meanwhile, mediated by a diauxie-inducible promoter, it is able to reduce the accumulation of FPP, a cytotoxic downstream product of GPP, during the exponential growth phase and mitigate the toxic effects of FPP on host cells while accumulating FPP and its downstream metabolites during the post-exponential phase [144]. Similarly, mediation by a temperature-sensitive promoter enables the separation of astaxanthin synthesis from cell growth and prevents the hindrance of cell growth by intermediate metabolites [80].

In general, the difficulties in constructing effective regulatory systems for xanthophyll production in model microorganisms to suit various industrial fermentation environments include the following: (1) the data on intracellular metabolic states in the xanthophyll-producing microbes are insufficient; (2) the metabolic pathway of xanthophyll is longer and more complex, which involves the coordination of multiple pathways for the synthesis and utilization of carbon sources, energy, cofactors, etc.; (3) finding the defects in metabolic networks based on metabolic state data and using genetic manipulation tools to design and construct regulatory modules or circuits that do not interfere with other necessary metabolic fluxes are challenging.

In the future, a database of metabolic networks for xanthophyll-producing microbes based on integrated multi-omics analysis and experimental validation needs to be established, along with efforts on the rational design of metabolic networks based on computer modeling and machine learning. The research on the metabolic network regulation of xanthophyll-producing strains is still at the beginning stage, and there is still huge room for improvement.

### 3.5. Selection of Microbial Hosts

At present, a variety of microorganisms have been developed as industrial microbial hosts. Prokaryotic microbial hosts mainly include *E. coli*, *B. subtilis*, etc., and yeast hosts mainly include conventional yeast *S. cerevisiae*, and non-conventional yeast *P. pastoris*, *Y. lipolytica*, *K. marxianus*, *C. glutamicum*, *Hansenula polymorpha*, *Candida maltosa*, etc. These microbial hosts have their own specific physiological and biochemical properties and different industrial applications fields. In this section, we mainly focus on *E. coli*, *S. cerevisiae*, *P. pastoris*, and *Y. lipolytica*, which have been reported as engineered hosts for xanthophyll synthesis. Their advantages and disadvantages for xanthophyll synthesis will be discussed.

#### 3.5.1. *E. coli*

Based on the abundance of genetic manipulation tools and its physiological and biochemical research foundation, *E. coli* is the most commonly used prokaryotic host for carotenoid metabolic engineering. By means of the lac operon, a simple multi-gene co-expression element, *E. coli* is the non-natural microbial host producing the most kinds of xanthophylls so far.

However, as a prokaryotic microorganism, *E. coli* has some inherent weaknesses in xanthophyll heterologous synthesis. (1) The efficiency of the MEP pathway in *E. coli* in synthesizing IPP and DMAPP, the precursors of all kinds of xantholylls, is lower than that of the MVA pathway in eukaryotic yeasts [17,145]. The IPP and DMAPP supply can be improved by introducing the MVA pathway into *E. coli*, but it also introduces an extra cell burden and metabolic crosstalk, which may affect the engineering host metabolic network and genetic stability [142]. (2) Since there are no subcellular organelles in *E. coli*, all reactions involving xanthophyll synthesis are located in the cytoplasm or cytoplasmic membrane. It is difficult to avoid the loss of precursors and intermediate metabolites, especially for the xanthophyll synthesis with a longer pathway and more cofactor requirements. (3) Almost all the enzymes in the xanthophyll synthetic pathway are membrane-related proteins. A low expression, misfolding, and inclusion body formation are common issues when a membrane protein is expressed in *E. coli* [146]. It had been thought that engineered *E. coli* produces higher levels of tetraterpenoids than yeast [145,147]. However, as research on engineering strategies has increased and intensified, the advantages of yeast have gradually emerged (Figure 2).

For the time being, *E. coli* is still the preferred host for testing the effectiveness of a xanthophyll synthesis pathway, but considering the high industrial production demand, eukaryotic model yeasts may have greater potential.

#### 3.5.2. *S. cerevisiae*

Contrary to the above shortcomings of *E. coli*, the MVA pathway of yeast itself can efficiently synthesize IPP, and organelle localization can improve the contact between the substrate and enzyme and reduce the loss of precursors and cofactors. A rich eukaryotic chaperone pool can help the membrane proteins to fold correctly. The common disadvantage of engineering yeast for xanthophyll synthesis may be that the transcription in a mono-cistronic manner is not conducive to multi-gene co-expression [148]. Moreover, different yeast species have different characteristics.

*S. cerevisiae* is currently the most common yeast species used in xanthophyll synthesis. As a conventional yeast, the strict homologous recombination of *S. cerevisiae* makes the genetic manipulation easier than other non-conventional yeasts [149]. Meanwhile, *S. cerevisiae* is the model strain for studying and applying multi-omics dataset mathematic analysis, CRISPR-based genome-scale engineering, GEMs (genome-scale metabolic models)-guided metabolic state prediction, etc. [150,151,152]. These systems’ biological strategies are expected to lead to a faster and more rational and intelligent design and optimization of the microbial host at a holistic level. Although these studies have not yet been used to engineer the xanthophyll-producing host, these advances provide lessons for *S. cerevisiae* to improve the xanthophyll production and host robustness. For other non-conventional yeasts, such as *P*. *pastoris*, *Y. lipolytica*, *K. marxianus* etc., the gene knock-down/out from the genome is more complex due to the non-homologous end joining (NHEJ) mechanism [153]. Although genome-editing tools such as CRISPR have been developed, they are still in their preliminary stages and represent a major technical barrier to the regulation of overall cellular metabolism levels. *S. cerevisiae* is a typical Crabtree-positive yeast that produces ethanol during glucose fermentation, which is generally considered to be unfavorable for the large-scale biosynthesis of compounds because of the waste of the carbon source and limited biomass formation [154]. This may be a point for the improvement of *S. cerevisiae* as a xanthophyll-producing strain. Other non-conventional yeasts in this review are all Crabtree-negative, which is their natural advantage as microbial hosts for the industrial synthesis of xanthophyll.

#### 3.5.3. *P. pastoris*

*P. pastoris* is a widely used heterologous protein expression chassis [155,156] and the mechanisms of protein expression regulation have been deeply explored. For example, the constitutive activation of the ER stress receptor Ire1p and the resulting UPR can help heterologous protein folding in *P. pastoris*, while the Ire1p is stress-induced in *S. cerevisiae* [157,158]. For xanthophyll synthesis, its powerful capability in membrane protein expression is a major advantage of *P. pastoris* over other yeast hosts [159].

In addition to the advantage in the protein expression aspect, *P. pastoris* is a methylotrophic yeast capable of growing on methanol as the only carbon source [160]. One-carbon metabolism is a key feature in future biorefinery [161], and it may also provide an inexpensive way to utilize carbon sources in the future industrial production of xanthophyll-engineered *P. pastoris*. *P. pastoris* is a Crabtree-negative yeast with no significant ethanol production during high-density fermentation, which comes with a higher flux of PPP and lower flux of glycolysis [162,163]. The higher flux of PPP allows for the production of more NADPH per carbon compared to *S. cerevisiae*, which may be more adapted to the demand of reducing power in xanthophyll synthesis. Another characteristic of *P. pastoris* is that the peroxisome volume can reach 80% of the cell volume under methanol- or oleate-induced conditions [148,164]. The advantages of peroxisomal localization mentioned in Section 3.2 may be better exploited in *P. pastoris*. Although there are no related reports yet, this unique advantage of *P. pastoris* is a potential research focus for high xanthophyll production.

Despite its many innate advantages, *P. pastoris* has been reported only as a host for astaxanthin, and the metabolic engineering of other xanthophyll and the mining of the advantages of peroxisome are barely reported.

#### 3.5.4. *Y. lipolytica*

In recent years, *Y. lipolytica* has shown rapid advances as an engineering host for xanthophyll. Its unique advantages in xanthophyll production mainly include: (1) its ability to utilize various waste media sources, such as vegetable hydrolysates, waste cooking oil, and even human urine [165]; (2) its inherently strong fatty acid metabolism, which gives it a larger pool of acetyl-coA and NADPH compared to other yeasts [165]; (3) the supersized LB theoretically provides an excellent site for xanthophyll synthesis and storage, although there are challenges in fully exploiting the advantages of this feature [130,166]. Regardless, the highest yields of β-carotene and astaxanthin occurred in *Y. lipolytica* (Figure 2). So far, the only reported xanthophylls synthesized with *Y. lipolytica* as the host are zeaxanthin and astaxanthin. Promisingly, the production of other xanthophylls with a high application value or very low yield, such as capsanthin, neoxanthin, etc., are expected to be further improved by relatively simple genetic manipulation.

The use of other yeast microbial hosts, such as *K. marxianus*, *C. glutamicum*, etc., for xanthophyll production has rarely been reported. The advantages of these hosts are yet to be further explored.

**Table 2:** Key enzymes with altered activity through amino acid mutations produced by directed evolution.

Key Enzymes	Natural Origin Species	Mutation Sites	Engineering Microbial Hosts	Effect on Activity	Ref.
ε-LCY	*Tagetes erecta*	F61N	*S. cerevisiae*	Enhanced δ-carotene formation by approximately 120%	[57]
		S401P		Decreased the activity	
CrtZ	*H. pluvialis* Flotow N-212	L288R	*S. cerevisiae*	Increased the astaxanthin production by ∼33%	[80]
	*B. vesicularis*	L53P	*Methylomonas* sp. *16a*	Increased the astaxanthin production	[167]
	*B. vesicularis*	F91S/V140G	*Methylomonas* sp. *16a*	Increased the astaxanthin production	[167]
BKT	*H. pluvialis* Flotow N-212	H165R/V264D/F298Y	*S. cerevisiae*	The canthaxanthin yield was increased approximately 2.4 folds	[81]
CrtW	*Brevundimonas* sp. SD212	A6T/T105A/L239M	*E. colli*	Improved astaxanthin production 5.35-fold	[104]
	*S. melonis*	F213L/R203W	*E. colli*	Improved the activity for converting cyclic hydroxylated intermediates into astaxanthin	[168] ^b^
		A215T			
		A205V			
		A208V			
		H96L			
CrtO ^a^	*R. erythropolis*	A190V	*Methylomonas* sp. *16a*	Increased the astaxanthin production	[167]

^a^ A kind of ketolase with a molecular weight greater than that of CrtW/BKT. ^b^ It also included additional mutations, as detailed in [168].

**Table 3:** Truncated key enzymes and their truncation positions.

Key Enzymes	Natural Origin Species	Truncated Length of N-Terminal (Amino Acid)	Engineering Microbial Hosts	Ref.
ε-LCY	*Marchantia polymorpha*	21 or 47	*S. cerevisiae*	[58]
CYP97A3	*Arabidopsis*	28 or 49	*E. coli*	[53]
CYP97C1	*Arabidopsis*	69	*E. coli*	[53]
BKT	*H. pluvialis* Flotow N-212	7	*S. cerevisiae*	[80]
ZEP	*Haematococcus lacustris*	59 and <100	*S. cerevisiae*	[96]
ZEP	*Arabidopsis thaliana*	57 and <100		
ZEP	*Solanum lycopersicum*	75 and <100		
CrtZ	*Haematococcus lacustris*	69		
CrtZ	*Solanum lycopersicum*	57		

**Table 4:** Subcellular locating signals of engineering microbial hosts.

Subcellular Organelle	Locating Signal	Engineering Microbial Hosts	Ref.
Endoplasmic reticulum	KDEL	*Y. lipolytica*	[84,169]
Mitochondria	MTS	*S. cerevisiae*	[121,170]
Peroxisome	PTS1	*Y. lipolytica, P. pastoris*	[84,169,171]
	Enhanced PTS1	*S. cerevisiae*	[124]
Lipid body	oleosin	*Y. lipolytica*	[84,169]
	Outer surface scaffold	*S. cerevisiae*	[172]
Yeast cell membrane	PM^SeV-C^	*S. cerevisiae*	[57]
*E. coli* cell membrane	GlpF	*E. coli*	[71]
	The signal peptide of OmpF and TrxA	*E. coli*	[114]

**Table 5:** The optimal copy number of xanthophyll pathway genes in a specific genetic background.

Xanthophyll	Key Enzymes	Optimal Copy Number	Engineering Microbial Hosts	Ref.
astaxanthin	CrtZ	3	*S. cerevisiae*	[82]
	BKT	3		
	CrtZ	2	*E. coli*	[71]
	CrtW	1		
canthaxanthin	BKT mutant	2	*S. cerevisiae*	[88]
lutein	CYP97A3	2	*S. cerevisiae*	[57]

## 4. Current Challenges and Future Prospects

Xanthophyll is a natural compound with rich biological activities and wide application fields. In recent years, due to the increasing demand of the market for natural xanthophyll and for the upgrading of traditional industrial production models of high-value compounds, the research on the production of this kind of compound using various model microbial hosts has developed rapidly. However, the xanthophyll produced by microbial metabolic engineering has not yet realized industrialization. Although the shift in the industrial production model of natural xanthophyll is related to a variety of factors, such as industrial inertia, the environment policy, etc., the most important is that the production costs of microbial engineering are not sufficient to cover the cost of the industrial model update. Therefore, the breakthrough in the xanthophyll production by engineering microbial hosts will still be a hot research topic in the future.

From the above factors and strategies that affect the production of xanthophyll, it can be concluded that future microbial metabolic engineering research on xanthophyll synthesis needs further efforts in the following aspects:

(1) The catalytic activity and substrate specificity of key enzymes of xanthophyll synthesis are usually low in the non-natural microbial host. At present, random mutation is still the main method for improving the activity and specificity of these enzymes. However, only one to two meaningful amino acid mutation sites can be obtained in each round of screening, so the efficiency and possibility of obtaining ideal mutants is very low. The three-dimensional structure of enzymes is an important basis for understanding their catalytic mechanism [173]. In terms of the three-dimensional structure, protein structure data on xanthophyll synthetic enzymes are extremely scarce. Various analytical methods for assessing the three-dimensional structures of proteins, such as X-ray diffraction [174], nuclear magnetic resonance (NMR) [175], cryo-electron microscopy (cryo-EM) [176], or the emerging Alphafold protein structure prediction tool [177,178], can be used to strengthen the knowledge of the structure and catalytic mechanism of these enzymes, which will provide important help for their rational design and for obtaining mutants adapted to the internal environment of host cells.

(2) The biochemical characteristics of xanthophyll itself result in a limited capacity of the host cell to accommodate it. Compared with carotene, xanthophyll, as an exogenous compound with a stronger antioxidant activity and relatively higher polarity, has a more detrimental effect on the physiological and biochemical status of the host [44]. Membrane localization or subcellular organelle localization of the xanthophyll metabolic pathway has been proven to improve its production, but the storage capacity for xanthophyll is also limited. The development of synthetic organelles brings hope for breaking through the storage limitations of xanthophyll in host cells. In *E. coli* and *S. cerevisiae*, synthetic organelles have been demonstrated for the functional localization of heterologous metabolic pathways [179,180,181]. In addition, cell-free systems also represent a promising technology suitable for future metabolic engineering applications [182,183]. Although the application scenarios for these technologies remain limited, it is theoretically feasible to customize the synthetic organelles suitable for the xanthophyll pathway and target produce storage, as well as use the cell-free system to dismantle various regulatory barriers within the host cell. Other strategies, such as increasing the xanthophyll solubility in the host by glycosylation modification to transfer it from the membrane to the cytoplasm to expand its storage space [184,185], or engineering specific transporters to facilitate xanthophyll secretion into the extracellular matrix [44,186], are also ways to bypass the accommodation capacity limitations of the host cell.

(3) The overall metabolic network in the host is not fully adapted to the efficient synthesis of xanthophyll. A longer xanthophyll synthesis pathway inevitably has an impact on the balance of the complex metabolic network of the host cell. At the same time, the metabolic network of the host does not match the efficient synthesis of xanthophyll due to the lack of natural screening and evolution. The metabolic engineering synthesis of xanthophyll is still in its beginning stage. The next step, using the systems and synthetic biology tools based on the combination of biotechnology and information technology and through the “design–build–test–learn” cycle [187] to achieve the rational design of xanthophyll high-yielding microbes, is an important direction for the metabolic engineering of xanthophyll and other compounds in the future.

(4) Various strategies were used piecemeal and could lead to the duplication of effort. In 2016, Shen et al. [46] reported that the introduction of the MVA pathway of *S. cerevisiae* into *E. coli* was able to increase the yield of zeaxanthin. In 2019, Takemura et al. [95] reported that the engineering of *E. coli* with violaxanthin synthesis was obtained by screening different sources of ZEP and oxygen reduction pairs. However, this study did not utilize the MVA introduction strategy described above. Similarly, in 2021 [103], the synthesis of capsanthin in *E. coli* was carried out independent of the previous synthesis strategies of zeaxanthin and violaxanthin. Because the synthesis pathway of various xanthophylls is a cascade reaction, different strategies should be comprehensively considered and combined. This is also a feasible way to improve the production of xanthophyll.

Besides the host cell metabolic engineering, the fermentation engineering optimization, bioavailability enhancement, product extraction, and packaging process improvement are also important issues in the process of xanthophyll industrialization. Among these, bioavailability is an important consideration for carotene and xanthophyll to exert health-protective effects. The bioavailability of carotenoid is low due to its instability and hydrophobicity, although that of xanthophyll is higher than that of carotene [188]. The bioavailability of xanthophyll is related to its stereoisomeric conformation, processing mode, and dosage form [189,190]. The feature associated with the metabolic engineering of xanthophyll synthesis is the conformation of the stereoisomer. The presence of chiral carbon in the molecular makeup of xanthophyll leads to the existence of multiple stereoisomers. For the lutein and zeaxanthin, the all-trans conformation is predominant in plants, and the bioavailability of the all-trans isomer may be higher than that of the cis-isomer [191,192]. For astaxanthin, the bioavailability of the cis-isomer was higher than that of the trans-isomer [193]. *H. pluvialis* and *Paracoccus carotinifaciens* produce cis-astaxanthin, whereas *X. dendrorhous* produces trans-astaxanthin [60]. The conformation of xanthophyll synthesized by metabolic engineering is determined by the stereoisomeric selectivity of exogenous enzymes. This is an aspect that needs to be considered in the metabolic engineering of xanthophyll.

In addition to the production of high-value products, microbial metabolic engineering for the uptake and utilization of cheap waste biomass is also developing rapidly [194]. Lignocellulose is one of the largest reserves of renewable energy. However, not all model microorganisms can directly utilize lignocellulose or its hydrolysates. Fortunately, engineered *E. coli* and *S. cerevisiae*, among others, have achieved the use of xylose, one of the main hydrolysis products of lignocellulose, as a carbon source for growth and metabolism [195]. Recently, by combining the xylose utilization pathway with the carotenoid synthesis pathway, *S. cerevisiae* and *K. marxianus* synthesized lycopene, carotene, and astaxanthin using xylose and glucose as carbon sources [196,197,198]. This opens the door for the construction of an energy-efficient and environmentally friendly xanthophyll-producing microorganism.

In conclusion, xanthophyll synthesis by the metabolic engineering of model microorganisms holds great industrialization prospects and is expected to revolutionize the industrial production mode of natural xanthophyll.

## Data Availability

No new data were used in this manuscript.
